# An orphan gene is necessary for preaxial digit formation during salamander limb development

**DOI:** 10.1038/ncomms9684

**Published:** 2015-10-26

**Authors:** Anoop Kumar, Phillip B. Gates, Anna Czarkwiani, Jeremy P. Brockes

**Affiliations:** 1 Institute of Structural and Molecular Biology, Division of Biosciences, University College London, London WC1E 6BT, UK; 2 Department of Genetics, Evolution and Environment, Division of Biosciences, University College London, London WC1E 6BT, UK

## Abstract

Limb development in salamanders differs from other tetrapods in that the first digits to form are the two most anterior (preaxial dominance). This has been proposed as a salamander novelty and its mechanistic basis is unknown. Salamanders are the only adult tetrapods able to regenerate the limb, and the contribution of preaxial dominance to limb regeneration is unclear. Here we show that during early outgrowth of the limb bud, a small cohort of cells express the orphan gene *Prod1* together with *Bmp2*, a critical player in digit condensation in amniotes. Disruption of *Prod1* with a gene-editing nuclease abrogates these cells, and blocks formation of the radius and ulna, and outgrowth of the anterior digits. Preaxial dominance is a notable feature of limb regeneration in the larval newt, but this changes abruptly after metamorphosis so that the formation of anterior and posterior digits occurs together within the autopodium resembling an amniote-like pattern.

## Abstract (web summary)

The first digits to form in salamanders are the two most anterior ones, unlike in other tetrapods. Here, the authors show that the gene *Prod1* is expressed during early outgrowth of the limb bud and is necessary for limb formation in salamanders.

## Graphical abstract

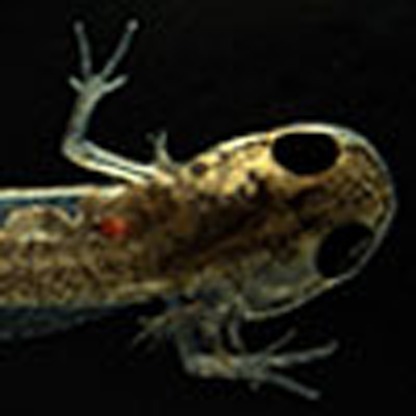

The patterning of skeletal elements during limb development in salamanders differs from other tetrapods (anuran amphibians and amniotes)^1^,^2^ in that it shows anterior or preaxial dominance in the order of formation and ossification of the zeugopodial and autopodial elements. In addition, some distal elements condense before more proximal ones^1^, and this is a departure from the familiar proximal to distal order of limb development in other tetrapods. Urodele limb development has been recognized as distinctive for over a 100 years^3^,^4^, and is widely regarded as an evolutionary novelty^1^,^5^. Although the timing of limb development shows considerable variation among salamander species^6^, it is invariant that the two most anterior digits are the first to form in larval salamanders. Amniotes and anurans show postaxial dominance where the posterior digits are first^7^,^8^,^9^. It has been noted that the limbs arise late in aquatic larval development in many salamanders, and there are significant functional demands on the developing limbs. In ponds or streams there is a requirement for anchorage, balance or movement, and this may be served by selective digital extension^6^,^10^. For example, in certain *Salamandrella* species the precocious outgrowth of the two preaxial digits is associated with the formation of a transient epidermal fin between them, and this is used in balance and locomotion^11^. In spite of these insights into the adaptive significance, there is no explanation for the mechanisms underlying the salamander-specific aspects of limb development. The expression of conserved genes implicated in tetrapod development, for example members of the *Hox A* and *D* clusters^12^,^13^, and sonic hedgehog^14^,^15^, has not suggested a compelling hypothesis.

The orphan gene *Prod1* was originally identified as retinoid-inducible gene in newt limb regeneration^16^, and is a member of the Three Finger Protein (TFP) family with a distinctive 12-residue α-helix in the third finger^17^. It has been implicated in the mechanism of regeneration^16^,^18^,^19^,^20^, and identified in nine different species (four families) of salamanders^21^. Extensive database searches and phylogenetic analyses have not revealed any TFP member that might correspond to a non-salamander Prod1^17^,^21^,^22^. We have tested the hypothesis that this orphan gene might be involved in salamander-specific aspects of limb development. Another feature of the salamander limb that is distinct from other adult tetrapods is the ability to regenerate, yet preaxial dominance has not been studied in the context of limb regeneration. In the newt, the most studied example of adult regeneration, the digits are found to regenerate together from the limb blastema^23^,^24^. Although there could be an underlying preaxial polarity, it is clearly difficult to analyse this property during adult regeneration.

We report here that *Prod1* is necessary for the presence of a small cohort of cells expressing the cytokine Bmp2 along with Prod1, and for formation of preaxial zeugopodial and autopodial elements. We find that in aquatic larval newts the initial extension of the two most preaxial digits is a notable feature of limb regeneration. This changes abruptly after metamorphosis so that regeneration of the eft-stage limb, as well as the adult, involves concerted extension of all four digits within the autopodium. This suggests that the mechanism of regeneration may be subject to selective pressure on transition from the aquatic to terrestrial environment. These data provide new insights into taxon-specific aspects of limb development and regeneration.

## Results

### Preaxial dominance and Prod1 during limb development

**Figure 1:**
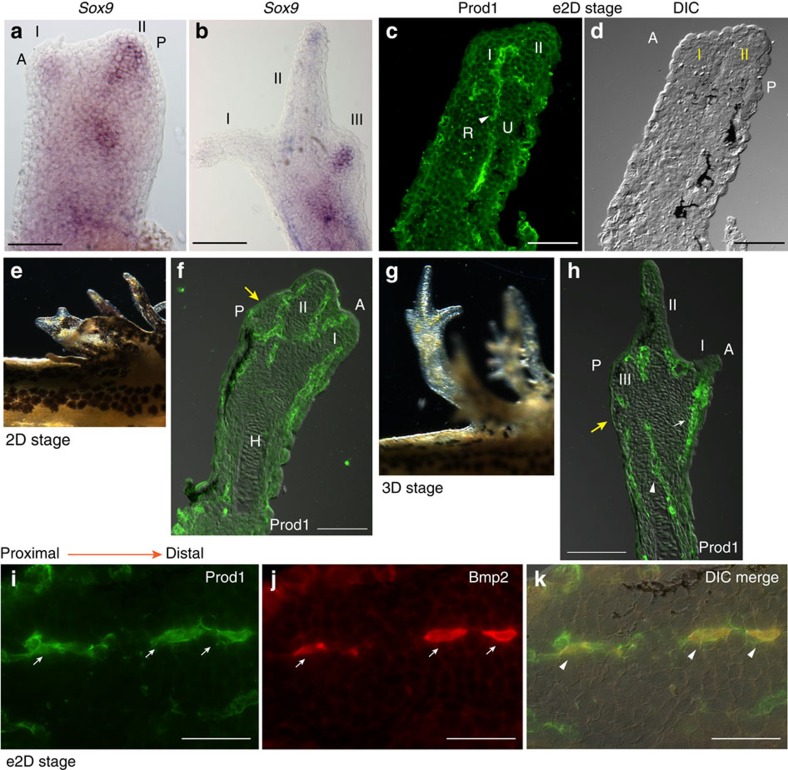
Emergence of preaxial dominance during limb development. (**a**) Expression of *Sox9* in digit primordia by RNA *in situ* hybridization at developmental stage e2D (*n*=6) and (**b**) at stage e3D (*n*=12). Scale bar, 100 μm. (**c**) Prod1 protein expression (arrowhead) in sections at e2D stage (*n*=12). (**d**) Morphology of the corresponding limb section (**c**) by differential interference contrast microscopy (DIC). Scale bar, 200 μm. (**e**) Larval limb at two-digit stage. (**f**) Prod1 protein expression in 2D stage limb. Presumptive digit three is arrowed (yellow; *n*=6). Scale bar, 200 μm. (**g**) Larval limb at 3D stage. (**h**) Prod1 protein expression at 3D stage. The connective tissue cells between radius/ulna (arrowhead), and cells in the differentiating dermis (arrowed white) are Prod1 positive. Presumptive digit four is arrowed (yellow; *n*=6). Scale bar, 200 μm. (**i**) Expression of Prod1 protein (arrowed) in a line of cells in longitudinal section of a limb at e2D stage. (**j**) Bmp2 protein (arrowed). (**k**) Composite DIC overlay. Scale bar, 50 μm. The arrowheads indicate cells expressing both Prod1 and Bmp2 protein (*n*=6). Developing digits are indicated I–III. A, anterior; P, posterior.

As noted for other salamander species^1^,^25^,^26^,^27^, the preaxial digits, II followed by I, extend precociously from the bud, and their primordia are identified by *in situ* hybridization with the early chondrogenic marker *Sox9* at the e2D stage^28^ (Fig. 1a, Supplementary Fig. 1). At the early three-digit (e3D) stage, digits I and II no longer react with the *Sox9* probe, while the primordium for digit III expresses this marker (Fig. 1b, Supplementary Fig. 1). Prod1 was detectable by antibody staining at e2D and was expressed initially in a line of cells, separating the presumptive radius and ulna, and bifurcating at its distal end at the location of the primordium of digit II (Fig. 1c,d). The development of zeugopodial and certain autopodial elements at the same stage has been noted in other salamander families^11^,^25^. At the 2D stage it is expressed in lines of cells between the digits (Fig.1e,f), and at the 3D stage it is found in the differentiating dermal cells and at the boundary between radius and ulna (Fig. 1g,h, Supplementary Fig. 2). The expression persists in the dermal cells through to adulthood^29^, and the dermis is an important source of cells for the regeneration blastema^30^,^31^. The specificity of Prod1 staining in the limb bud was confirmed by using a second antibody to a non-overlapping epitope of the protein, and this gave comparable results (Supplementary Fig. 2). The distribution of Prod1 staining was reminiscent of earlier studies of *Bmp2* expression in the developing axolotl limb bud by *in situ* hybridization^32^, and we obtained similar results in the newt using antisense probes for *Prod1* and *Bmp2* (Supplementary Fig. 3). *Bmp2* is a cytokine that is considered critical for digit formation in amniotes, and that activates *Sox9* expression in this context^33^,^34^. To investigate co-expression at a single-cell level, we made an antibody to newt Bmp2 that allowed dual fluorochrome detection in sections of the limb bud. The lines of Prod1-positive cells showed clear examples of co-expression with Bmp2 (Fig. 1i–k, Supplementary Fig. 3).

### Disruption of Prod1 during limb development

**Figure 2:**
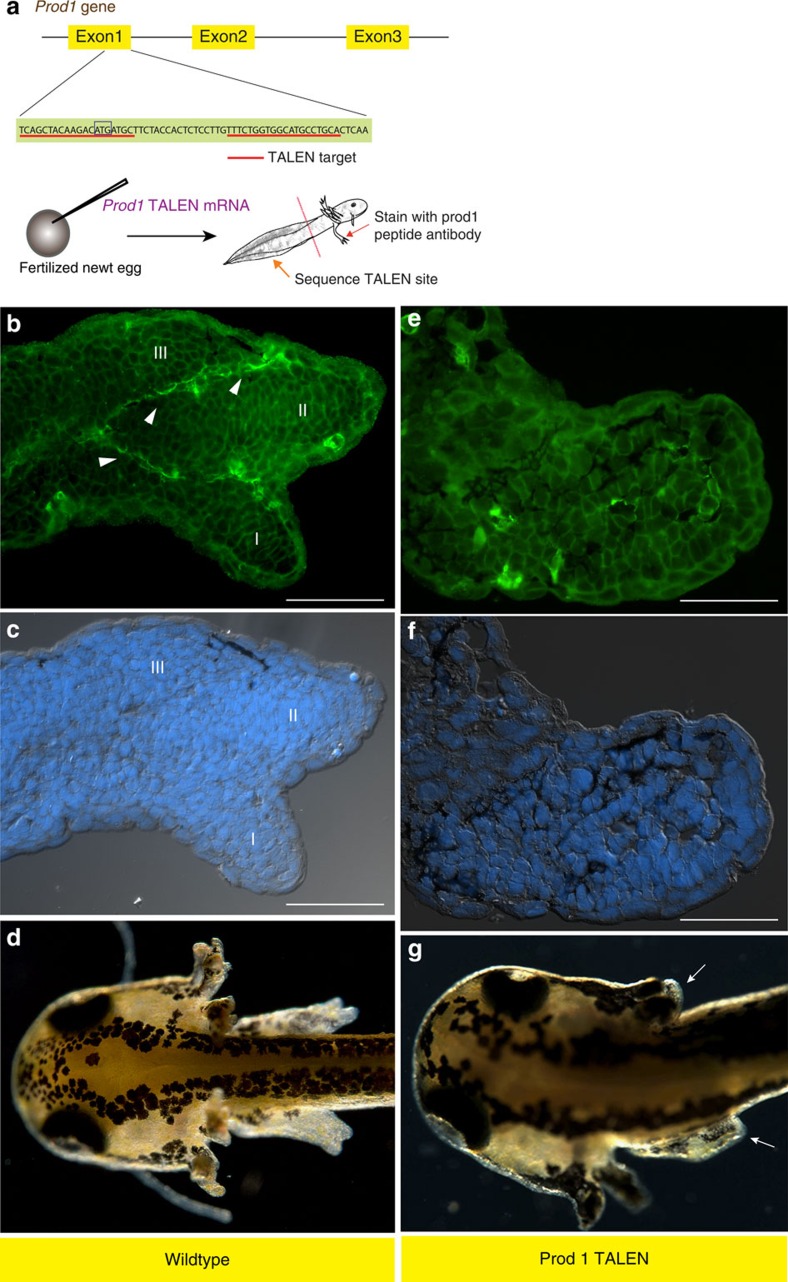
Disruption of *Prod1* abrogates axial patterning of the limb. (**a**) Schematic diagram illustrating TALEN design and workflow. The efficiency of expression of the injected mRNA is 53% (*n*=62/117) at 24 h post-injection using GFP mRNA. (**b**) Prod1 protein expression (arrowhead) in e3D stage limb. (**c**) Limb section with nuclear staining and differential interference contrast (DIC) overlay. (**d**) Wild-type larval morphology. (**e**) Prod1 protein reactivity is absent in the limb tissues. (**f**) Morphology by nuclear staining and DIC overlay (**g**) Larval morphology showing growth-arrested limb bud (arrowed). R, radius; U, ulna; H, humerus. Digits, I–III. Scale bar, 100 μm.

**Figure 3:**
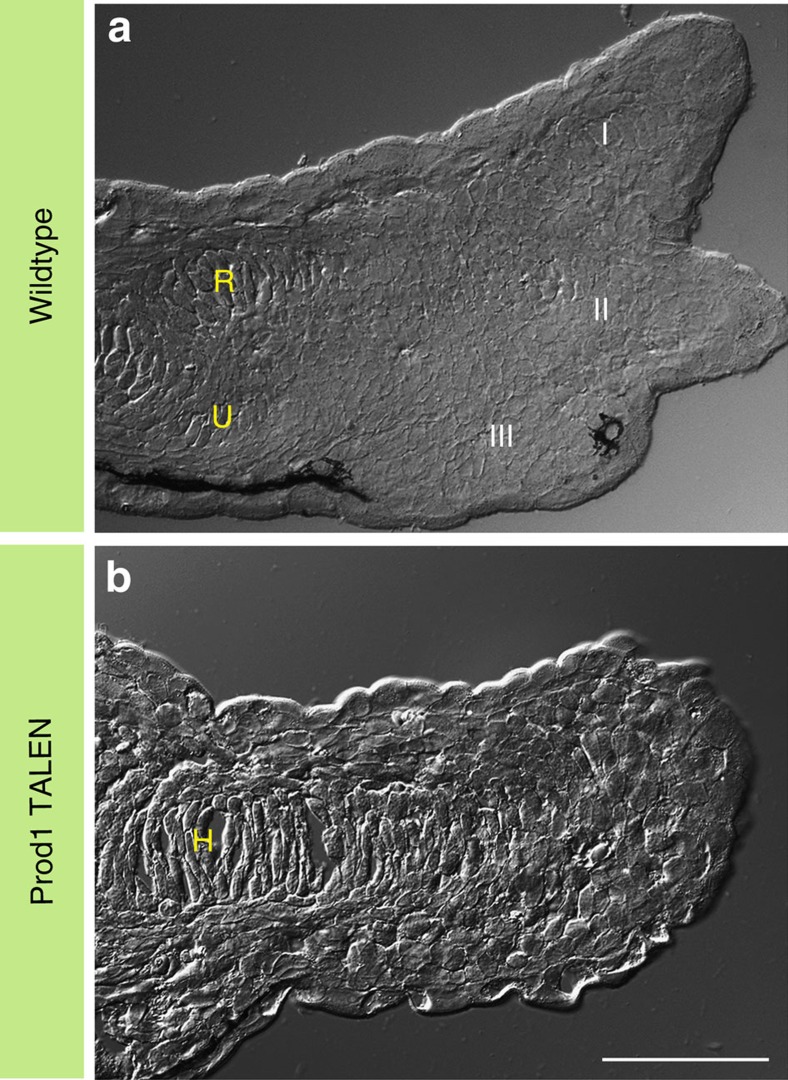
Morphology of the limb. Serial longitudinal section of a limb showing morphological features using differential interference contrast (DIC) microscopy. (**a**) Section of a wild-type limb at e3D stage. (**b**) A section showing morphology of the limb bud in a Prod1-negative larva. Note the absence of distal zeugopodial and autopodial elements. R, radius; U, ulna; H, humerus. Digits, I–III. Scale bar, 100 μm.

The functional significance of Prod1 expression in the limb bud was evaluated by injecting fertilized newt eggs with two synthetic mRNAs encoding TALEN monomers directed at a sequence in exon 1 of the *Prod1* gene close to the start of translation (Fig. 2a). The *Prod1* genes from individual larvae that developed to the limb bud stages were sequenced by nested PCR to determine if the TALEN target site was disrupted, and the limbs were analysed by antibody staining for Prod1 expression. In cases where the exon 1 sequence was intact, Prod1 staining was detected in characteristic lines of cells in limb buds that had progressed to the e3D stage (Fig. 2b–d). We identified 17 cases where the target site was disrupted, and three were analysed by subcloning to identify the indel mutations generated (Supplementary Fig. 4). Fifteen (of 17) were negative for Prod1 expression in the limb bud, and all were arrested before zeugopodial development, and without condensation of digits II and I (Fig. 2e–g), having developed normally to this point. Analysis of serial limb sections by differential interference contrast microscopy has confirmed that the normal e3D stage larvae had cartilage elements associated with the developing digits, and the radius/ulna (Fig. 3a). In the Prod1-negative disruptants, the humerus was visible but there were no elements associated with radius/ulna development or digit formation (Fig. 3b). The absence of Prod1 staining in the limb bud was also associated with a loss of Bmp2-positive cells in the characteristic lines (Supplementary Fig. 5). Two cases (of 17) were positive for Prod1 expression in the limb bud, due to mosaic expression in the disrupted larvae, and both cases progressed to the e3D stage. We conclude that Prod1 expression is necessary to generate the lines of cells expressing Bmp2, and to induce zeugopodial development and formation of the two preaxial digits.

### Digit patterning and regeneration

**Figure 4:**
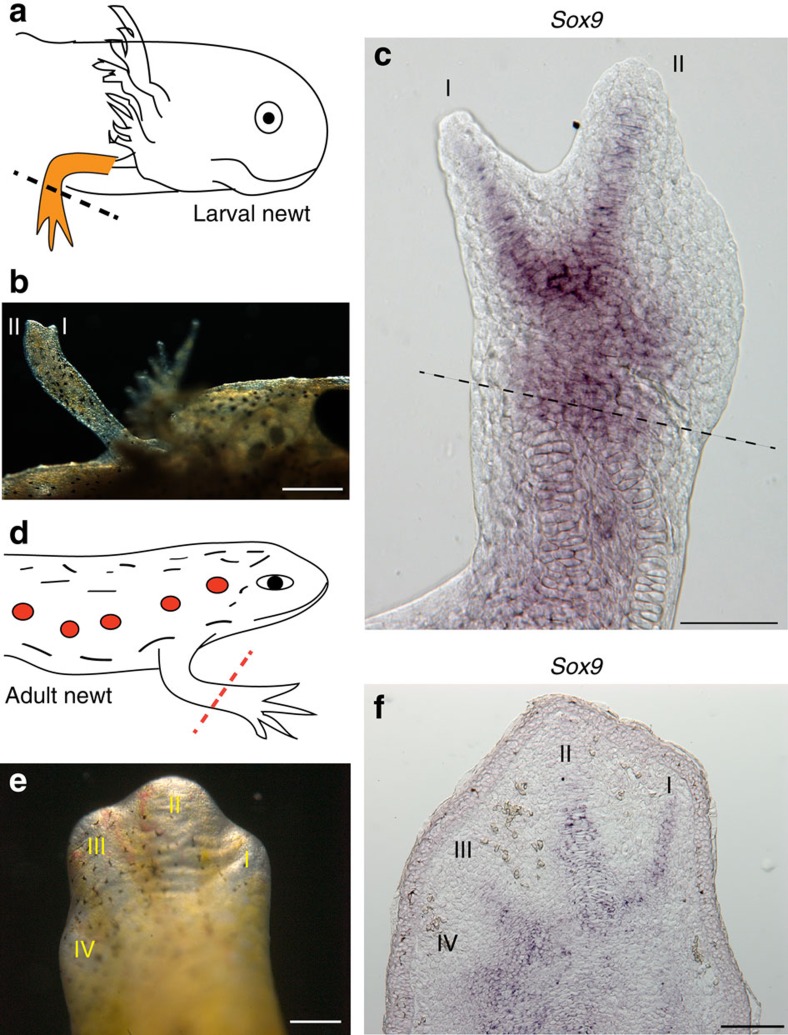
Digit patterning during limb regeneration. (**a**) Schematic diagram of the procedure in larval newts. 3D stage larval limbs were amputated at mid-zeugopodium and allowed to regenerate (*n*=6). (**b**) Larval limb regeneration at 12D. Scale bar, 0.5 mm. (**c**) Whole-mount RNA *in situ* hybridization of the 12D limb regenerate showing *Sox9* expression in preaxial digits I and II. Dotted line indicates the level of amputation. Scale bar, 200 μm. (**d**) Schematic diagram of the procedure in adult newts. The forelimb was amputated at mid-zeugopodium (*n*=12). (**e**) A representative regenerating limb at day 30. Scale bar, 200 μm. (**f**) RNA *in situ* hybridization showing *Sox9* expression in a longitudinal section of an early four-digit limb regenerate. Scale bar, 200 μm. Digits, I–IV.

In view of the involvement of Prod1 in limb regeneration^16^,^19^,^20^,^29^, we analysed the contribution of preaxial dominance to this process in larval and adult newts. In the larvae, transection of three digit, or fully differentiated four-digit pre-metamorphic limbs evoked formation of a blastema, and the initial extension of digits II and I (Fig. 4a,b). There was expression of *Sox9* in these digits before the appearance of digit III (Fig. 4c), as noted earlier for limb development (Fig. 1a). Most of the cells in the larval blastema expressed Prod1 on their surface (Supplementary Fig. 6), as described for adult newt limb regeneration^16^, whereas in limb development only sporadic cells in the early bud were positive at stages before e2D. At the stage of digit extension during regeneration, Prod1 was expressed in the mesenchymal cells adjacent to the cartilage elements of digits II and I (Supplementary Fig. 6).

**Figure 5:**
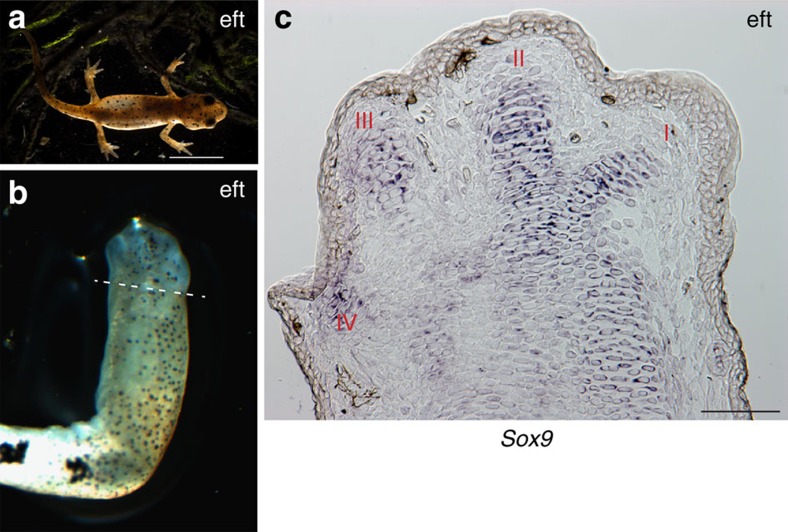
Digit patterning in post-metamorphic newts. (**a**) A captive bred newt (eft) approximately 3 months of growth after hatching. Scale bar, 0.5 cm. (**b**) Regenerating limb of an eft at 32D post-amputation. Dotted line depicts the level of limb amputation (*n*=4). (**c**) RNA *in situ* hybridization showing *Sox9* expression in regenerating digits of the corresponding limb (**b**). Scale bar, 100 μm. Digits, I–IV.

The occurrence of preaxial dominance in larval limb regeneration was noteworthy, because earlier descriptions of adult regeneration in the newt have found that the forelimb digits extend together^23^,^24^. An image of a regenerating limb with digits extending at the ‘paddle' stage was reminiscent of autopodial development in amniotes (Fig. 4d,e), as well as in terrestrial plethodontid salamanders^25^,^35^. The analysis of *Sox9* expression at an earlier stage of adult regeneration shows that all four digits are labelled, although I and II remain more prominent (Fig. 4f) than the postaxial III/IV. There is apparently a transition between the larval preaxial version, and the more concerted mechanism of digit extension from the adult blastema. This change might occur at metamorphosis, or later when the limb approaches adult size. We maintained larval newts in the laboratory until they underwent metamorphosis and lost the gills to give rise to the stage called an eft^36^ (Fig. 5a). Transection of the eft limb evoked an adult version of regeneration with co-extension of digits I–IV (Fig. 5b), and co-expression of *Sox9* (Fig. 5c, Supplementary Fig. 7), thus implicating metamorphosis in the transition. We analysed this aspect of limb regeneration in the paedomorphic axolotl (*Ambystoma mexicanum*) and found that although the external appearance of digit extension was less obvious than the larval newts, *Sox9* expression showed clear labelling of the primordia for preaxial digits I and II in advance of postaxial digits (Supplementary Fig. 8). This suggests that unlike post-metamorphic newts, preaxial dominance operates at the level of digit formation in the paedomorphic axolotls.

## Discussion

It is noteworthy that loss of Prod1 does not affect embryonic, larval or limb development before the stage of condensation of the radius/ulna and digits I and II. Disruption of the *Prod1* gene leads to loss of the cells expressing Bmp2 along with Prod1. *Bmp2* has recently been proposed, along with *Sox9*, as a key player in a reaction–diffusion network underlying the concerted formation of digits in amniotes^37^. This mechanism seems somewhat problematic in view of the timing of events in the salamander limb bud: there is a 5–7-day gap between the appearance of digits II/I and digit III, and in the appearance of *Sox9* expression corresponding to the primordia (Fig. 1b). It remains possible that there could be separate episodes of the reaction–diffusion process. The loss of Prod1 does not result in a default postaxial option resembling amniote limb development, and in view of the loss of Bmp2 expression, this would not be expected.

The differences in urodele development may have evolved in relation to the functional demands on the limb of an aquatic larva, as discussed earlier. The free-living aquatic larva is considered by most investigators to be the ancestral condition for the amphibian lineages^38^,^39^,^40^. It is possible that these same pressures led to selection for the limb to regenerate after biting or injury^41^. Our results show that preaxial dominance of digit formation is incorporated as part of the mechanism of limb regeneration in larval newts and axolotls. It is striking that this aspect changes after metamorphosis, so that the concerted formation of the digits in a regenerating eft or adult newt limb resembles digit development in terrestrial plethodontid salamanders^25^,^35^, anuran frogs^7^,^9^ and amniotes^8^,^33^. Our finding of this post-metamorphic transition is consistent with the view that selection operates on regeneration, as well as on other established terrestrial adaptations like the mechanisms of feeding and hearing^10^,^42^.

*Prod1* has been identified in two species of early branching salamanders of the family Hynobiidae^21^,^43^, and was therefore likely to have been present in the last common ancestor of crown group salamanders at the beginning of the Jurassic. Evidence for the occurrence of preaxial dominance, and for limb regeneration, has recently been reported in fossils of dissorophoid temnospondyl larval amphibians that lived in mountain lakes of the early Permian^44^,^45^,^46^. This is particularly direct and compelling for preaxial dominance of the branchiosaurid *Apateon*^46^, where the sequence of ossification in the limb skeleton can be seen in multiple examples in a developmental series. The evidence for limb regeneration^45^ is more indirect and has come from characteristic limb abnormalities in specimens of the dissorophoid temnospondyl *Micromelerpeton.* The precise relation of temnospondyls to crown group salamanders is a matter for conjecture^44^,^47^, but these two aspects, preaxial dominance and regeneration, may have evolved together and it remains possible that Prod1 was present in these species.

## Methods

### Animals, embryo collection and procedures

Adult newts (*Notophthalmus viridescens*) were obtained from Charles D. Sullivan & Co., TN, USA. The embryo injection experiments were performed during the breeding months December–May, spanning over 3 years. The newts were maintained at 18 °C in groups of 25–30 per aquarium with aquarium plants of the *Vallisneria* species. To induce spawning, the female newts were injected with Chorulon (30 IU per newt) on alternate days for 7–10 days. They were fed liberally with live blood worms throughout the period.

Newt larvae were raised as described with modifications^48^. The larvae were maintained in custom-fabricated chambers at 22–24 °C in a modified LMS incubator with 12L:12D photoperiodic cycle. Metamorphosis occurred naturally among the larval animals approximately 70 days post-hatching. The efts were maintained on a diet of live blood worms and *Daphnia.*

Juvenile axolotls (10–12 cm) were purchased from local suppliers. The animals were fed with live blood worms and maintained at 22 °C throughout the experimental period.

For regeneration experiments, larval and adult newts were anaesthetized in 0.05–0.1% Tricaine (Sigma) in 0.25% Holtfreter's solution. Pre- and post-operative care were taken according to the guidelines approved by the Home Office, UK. The limb transection in newts and axolotls was performed at mid-zeugopodium. In eft stage animals, the limbs were amputated during the climax stage of metamorphosis and the animals were allowed to regenerate through the transition phase to the eft stage.

### Synthesis of Prod1 TALEN constructs

Two pairs of TALEN constructs, L1/R1 and L2/R2, corresponding to target sites in exon 1 and exon 2, respectively, were synthesized in the vector GT-EN-12020-2 (GeneCopoeia). The TALEN containing *aseI* fragment was sub-cloned into pBSK vector (Stratagene). These were first linearized with *xbaI* and then capped and polyadenylated mRNA was synthesized using mMESSAGE mMACHINE T7 ULTRA Transcription Kit (Life Technologies). The mRNA aliquots were stored at −70 °C until use. No gene disruptants or Prod1-negative limb buds were obtained after injections of L2/R2 and this combination was not pursued further.

### Embryo injection and generation of newt larvae

Single- or two-cell stage embryos were injected with 750 pg TALEN mRNA in a total volume of 3 nl containing phenol red as described^49^ with minor modifications. The embryos were injected without dejellying. Control embryos were injected with L1, R1 or R2 TALENs alone at the same concentration. The embryos were raised at 24 °C and staged as described^28^. Phenotypes of the developing limb were assessed at various stages of development, and the end point of the experiment was determined as the wild-type control larvae reached e3D. Phenotypic abnormalities of the developing limb bud (growth arrest at limb bud stage) were assessed in normal developing and control TALEN injected embryos from various batches. In control embryos (no injection) that developed to the limb bud stage, 1% (3/296) were arrested before forming digits, while in control TALEN-injected embryos the number was 1.3% (2/150). In embryos injected with TALEN mRNAs directed to the exon 1 site, the number was 41% (109/266).The phenotypes of the control and Prod1 TALEN-arrested larvae were digitally recorded, and the limbs were dissected and processed for immunofluorescence. The trunk segments of the larvae were frozen immediately for analysis by genomic PCR.

### Mutation analysis

DNA was extracted from the tissue using DNeasy Blood & Tissue Kit (Qiagen). Prod1 sequences were rescued by two rounds of PCR using *PfuUltra* High-Fidelity DNA polymerase (Stratagene). Genomic DNA (500 ng) was first amplified with primers (Forward 5′-AGCAATGAGGCTCGTTAGGT-3′, Reverse 5′-ATCTGGTCTATGGCTAGGAG-3′) for 30 cycles. This was diluted 1:50 and 1 μl was amplified in 50 μl reaction volume for 30 cycles with primers (Forward 5′-GCATGGCTGACGTCACTGTT-3′, Reverse 5′-GAGGGTGTCATTGTGCTACA-3′). The resulting Prod1 DNA was purified using QIAquick PCR Purification Kit (Qiagen), and sequenced with primer 5′-CTTTGGCACTGCTGCGTGTA-3′ (Source BioSciences). Mutant examples were identified by the presence of multiple sequence traces at the TALEN cleavage site. To determine the precise nature of the lesions, selected samples from the rescued Prod1 DNA was blunt-end ligated into the *smaI* site of pBSK vector and individual clones were grown and sequenced.

### Whole-mount *in situ* hybridization and optical clearing

Synthesis of the riboprobes for the detection of Prod1 has been described^16^. On the basis of the *Pleurodeles waltl Sox9* sequence^50^, we designed oligos (Forward 5′-AGCTCTGGAGGCTGCTGAATGA-3′, Reverse 5′-CTGTAGTAGGTGTTAGAGCTCT-3′) to amplify the newt orthologue from a newt blastema cDNA library. We cloned this 900-bp fragment into the vectors pBSK (Stratagene) and pCI-neo (Promega). To make an antisense probe, the construct was linearized with *xhoI* and T3 polymerase was used for riboprobe synthesis. For a sense probe the vector was linearized with *xbaI* and riboprobe was synthesized with T7 polymerase. To generate probe specific to *Bmp2*, oligos (Forward 5′-CGCCTCGAGGTTTTGGAAGGCCTGCCAGA-3′, Reverse 5′-GCGTCTAGAAGTCTGCACAATGGCGTGGT-3′) were used to amplify a 700-bp *Bmp2* fragment from newt blastema cDNA library. This fragment was cloned into the *xhoI* and *xbaI* sites of pBSK and pCI-neo vectors. To make an antisense probe, we linearized the construct with *xhoI* and used T3 polymerase, and for a sense probe the vector was linearized with *xbaI* and we used T7 polymerase for probe synthesis. To assess gene expression, the respective pCI-neo expression vectors were transiently transfected into HEK293 cells and analysed with the corresponding riboprobes by *in situ* hybridization. The antisense riboprobes, *Prod1*, *Sox9* and *Bmp2* gave clear signals, whereas, none of the control sense probes gave any detectable signal after parallel incubation.

The *in situ* hybridization protocol was based on the methods described^51^,^52^ with modifications. The larval newts were killed by overdose of Tricaine, washed in PBS and fixed in 4% paraformaldehyde (PFA) overnight at 4 °C. The samples were washed in PBST (PBS/0.1% Tween-20) and bleaching of the pigments were performed in 0.5% KOH containing 6% hydrogen peroxide for 10 min under a stereomicroscope with cold light source. The larvae were washed again in PBST and post-fixed in ice-cold methanol at −20 °C for 10 min. The tissues were rehydrated in descending grades of MeOH:H_2_O (75:25; 50:50; 25:75) and finally in PBST (2 × ). Proteinase-K (recombinant PCR grade; Roche) treatment was carried out at room temperature at 40 μg ml^−1^ in PBST for 10 min. The samples were post-fixed in 4% PFA for 20 min and washed twice in PBST and acetylated for 5 min. The larvae were washed again in PBST and finally in PBS. The limb tissues were dissected and mounted on Superfrost Plus slides containing the prewarmed (55 °C) hybridization solution. Hybridization was performed with the respective riboprobes at 55 °C for 18–20 h. After hybridization, the samples were transferred to Costar Netwell inserts in 12-well microplates and washed extensively in PBST containing 0.1% CHAPS. The tissue samples were blocked in 10% sterile filtered heat-inactivated goat serum for 1 h and incubated overnight in Roche blocking solution containing DIG-labelled antibody (1:2,000) at 4 °C. Tissue samples were extensively washed in PBST, and finally in Alkaline Phosphatase buffer. BCIP/NBT (Promega) colour reaction solution was prepared in Alkaline Phosphatase buffer, and limb tissues were incubated at room temperature and monitored periodically. At the end of colour development (6–8 h or overnight), the samples were washed briefly in PBST and reaction was terminated by fixation in 4% PFA. The samples were washed again in 1% PBST and transferred to optical clearing solution.

Optical clearing of the tissues were performed using a modified Scale^53^. The clearing agent consists of 4 M Urea containing 0.5% Triton-x100, pH 7.5 (adjusted with 1 N NaOH), and is stored at room temperature. The tissues were transferred to the clearing solution and incubated at room temperature for 24–48 h with gentle shaking. The samples were observed under a stereomicroscope and cleared tissues were washed in 0.1% PBST for several hours with occasional changes and finally washed in PBS and equilibrated in Vectamount (Vector Laboratories) aqueous mounting medium. The limb samples were mounted on a Superfrost Plus slide and cover slipped for observation. The clearing agent does not appear to interfere with BCIP/NBT reaction product.

### Section *in situ* hybridization

The limb tissues were collected and fixed in 4% PFA overnight at 4 °C. The tissues were washed in PBS, infiltrated with 20% sucrose and embedded in Tissue Tek-II. Serial longitudinal sections of 16 μm were cut, mounted on Superfrost Plus slides and air dried for 2 h at 55 °C before hybridization. *In situ* hybridization was performed essentially as described in the above section, except that the concentration of Proteinase-K was reduced to 10 μg ml^−1^ for newt tissues, or 5 μg ml^−1^ for axolotl, with incubation for 12 min. Typically, the colour development occurred overnight at room temperature.

### Antibodies and immunostaining

The two affinity-purified Prod1 antibodies 683 and 684 have been described previously^16^,^29^. The available newt *Bmp2* sequence was extended using SMARTer RACE 5′/3′ Kit (Clontech). 5′ race was carried out using oligo 5′-CAGGCCTTCCAAAACTTCTTCGTGGTGGAA-3′ and 3′ RACE with oligo ACTCCACCAACCACGCCATTGTGCAGACTT. Newt Bmp2 peptide antibodies 336 (FRELVEDSSERNSSNL) and 337 (QRHVRVRRSAHQDEYS) were raised in guinea pigs by Eurogentec. The newt Bmp2 lacks the N-terminal secretory signal sequence; therefore, an axolotl-newt chimeric construct was assembled in the *xhoI*, *notI* sites of pCI-neo expression vector by amplifying axolotl cDNA with oligos (Forward 5′-CCCAGCTCGAGCTTCCTGAA-3′, Reverse 5′-GAGGTCTAGACCCTGGCCCTGGTA-3′), whereas, newt cDNA was amplified with oligos (Forward 5′-CAGGATCTAGACCTGCGACA-3′, Reverse 5′-TATAAGCGGCCGCTTGGTCTCTAATGGCACCCA-3′). The chimeric vector was transiently expressed in HEK293 cells, and *Bmp2* expression was analysed 48 h after transfection, using antibodies (336 and 337) along with concentration-matched guinea pig IgG antibodies in parallel. The antibody 336 at 1:400 dilution gave clear reactivity in HEK293 cells, and was used in analysis of the limb sections by immunostaining.

In all cases, the limb tissues were fixed overnight in 4% PFA at 4 °C, washed in PBS, equilibrated in 20% sucrose and embedded in Tissue Tek-II. Serial longitudinal sections were cut at 12 μm in a Leica CM 1850 cryostat, mounted on Superfrost Plus slides and stored at −20 °C until processing. The slides were air dried, rehydrated in PBS/Tx-100 (0.01%) and blocked with 10% heat-inactivated goat serum containing 0.1% Roche western blot blocking solution. The slides were incubated overnight with primary antibodies at 4 °C, washed in PBS and incubated in secondary antibodies for 1 h. For double antibody incubations, the primary antibodies were incubated together, whereas, the secondary antibody incubation was performed individually.

### Microscopy

The slides from whole-mount and section *in situ* hybridization were observed under a Zeiss Axio Zoom.V16 microscope and the images were captured by AxioCam 506 or Axiocam HRc colour digital camera using Zeiss Zen imaging suite. In many cases, image stacks were acquired and processed using the Extended Depth of Field module to generate in-focus images of whole limbs. Tissue sections reacted with antibodies were observed under a Zeiss Axioskop2 microscope and images were acquired using Hamamatsu Orca ER camera controlled through Openlab software. The contrast settings for the images were applied globally, and the images were processed and assembled using Adobe CS6 suite (Adobe Systems).

## Additional information

**How to cite this article:** Kumar, A. *et al.* An orphan gene is necessary for preaxial digit formation during salamander limb development. *Nat. Commun.* 6:8684 doi: 10.1038/ncomms9684 (2015).

## Supplementary Material

Supplementary Information Supplementary Figures 1-8

## References

[b1] Frobisch N. B. & Shubin N. H. Salamander limb development: integrating genes, morphology, and fossils. Dev. Dyn. 240, 1087–1099 (2011) . 21465623 10.1002/dvdy.22629

[b2] Shubin N. H. & Alberch P. in Evolutionary Biology Vol. 20, eds Hechht M. K., Wallace B., Prance G. I. 319–387Plenum Press (1986) .

[b3] Holmgren N. On the origin of the tetrapod limb. Acta Zool. 14, 185–295 (1933) .

[b4] Schmalhausen J. J. Die entwicklung des extremitatenskelettes von *Salamandrella keyserlingii*. Anat. Anz. 37, 431–446 (1910) .

[b5] Shubin N. H. Origin of evolutionary novelty: examples from limbs. J. Morphol. 252, 15–28 (2002) . 11921033 10.1002/jmor.10017

[b6] Shubin N. & Wake D. in Biology of the Amphibia ed Heatwole H. 1782–1808Surrey Beatty and Sons (2003) .

[b7] Kerney R. & Hanken J. Gene expression reveals unique skeletal patterning in the limb of the direct-developing frog *Eleutherodactylus coqui*. Evol. Dev. 10, 439–448 (2008) . 18638321 10.1111/j.1525-142X.2008.00255.x

[b8] Zhu J. *et al.* Uncoupling Sonic hedgehog control of pattern and expansion of the developing limb bud. Dev. Cell 14, 624–632 (2008) . 18410737 10.1016/j.devcel.2008.01.008 PMC8284562

[b9] Trueb L. & Hanken J. Skeletal development in *Xenopus laevis* (Anura: Pipidae). J. Morphol. 214, 1–41 (1992) . 1433306 10.1002/jmor.1052140102

[b10] Duellman W. E. & Trueb L. Biology of Amphibians McGraw-Hill, Inc. (1986) .

[b11] Vorobyeva E. I. & Hinchliffe J. R. Developmental pattern and morphology of *Salamandrella keyserlingii* limbs (Amphibia, Hynobiidae) including some evolutionary aspects. Russ. J. Herpetol. 3, 68–81 (1996) .

[b12] Gardiner D. M., Blumberg B., Komine Y. & Bryant S. V. Regulation of *HoxA* expression in developing and regenerating axolotl limbs. Development 121, 1731–1741 (1995) . 7600989 10.1242/dev.121.6.1731

[b13] Torok M. A., Gardiner D. M., Shubin N. H. & Bryant S. V. Expression of *HoxD* genes in developing and regenerating axolotl limbs. Dev. Biol. 200, 225–233 (1998) . 9705229 10.1006/dbio.1998.8956

[b14] Imokawa Y. & Yoshizato K. Expression of *Sonic Hedgehog* gene in regenerating newt limb blastemas recapitulates that in developing limb buds. Proc. Natl Acad. Sci. USA 94, 9159–9164 (1997) . 9256452 10.1073/pnas.94.17.9159 PMC23086

[b15] Torok M. A., Gardiner D. M., Izpisua-Belmonte J. C. & Bryant S. V. *Sonic Hedgehog (shh)* expression in developing and regenerating axolotl limbs. J. Exp. Zool. 284, 197–206 (1999) . 10404648

[b16] Morais da Silva S. M., Gates P. B. & Brockes J. P. The newt ortholog of CD59 is implicated in proximodistal identity during amphibian limb regeneration. Dev. Cell 3, 547–555 (2002) . 12408806 10.1016/s1534-5807(02)00288-5

[b17] Garza-Garcia A., Harris R., Esposito D., Gates P. B. & Driscoll P. C. Solution structure and phylogenetics of Prod1, a member of the three-finger protein superfamily implicated in salamander limb regeneration. PLoS ONE 4, e7123 (2009) . 19771161 10.1371/journal.pone.0007123 PMC2740830

[b18] Brockes J. P. & Gates P. B. Mechanisms underlying vertebrate limb regeneration: lessons from the salamander. Biochem. Soc. Trans. 42, 625–630 (2014) . 24849229 10.1042/BST20140002

[b19] Echeverri K. & Tanaka E. M. Proximodistal patterning during limb regeneration. Dev. Biol. 279, 391–401 (2005) . 15733667 10.1016/j.ydbio.2004.12.029

[b20] Kumar A., Godwin J. W., Gates P. B., Garza-Garcia A. A. & Brockes J. P. Molecular basis for the nerve dependence of limb regeneration in an adult vertebrate. Science 318, 772–777 (2007) . 17975060 10.1126/science.1147710 PMC2696928

[b21] Geng J. *et al.* Identification of the orphan gene *Prod1* in basal and other salamander families. Evodevo 6, 9 (2015) . 25874078 10.1186/s13227-015-0006-6 PMC4396064

[b22] Garza-Garcia A. A., Driscoll P. C. & Brockes J. P. Evidence for the local evolution of mechanisms underlying limb regeneration in salamanders. Integr. Comp.Biol. 50, 528–535 (2010) . 21558221 10.1093/icb/icq022

[b23] Iten L. E. & Bryant S. V. Forelimb regeneration from different levels of amputation in the Newt, *Notophthalmus viridescens*: length, rate, and stages. Wilhelm Roux' Archiv. 173, 263–282 (1973) . 10.1007/BF00575834 28304797

[b24] Vlaskalin T., Wong C. J. & Tsilfidis C. Growth and apoptosis during larval forelimb development and adult forelimb regeneration in the newt (*Notophthalmus viridescens*). Dev. Genes Evol. 214, 423–431 (2004) . 15322877 10.1007/s00427-004-0417-1

[b25] Franssen R. A., Marks S., Wake D. & Shubin N. Limb chondrogenesis of the seepage Salamander, *Desmognathus aeneus* (Amphibia: Plethodontidae). J. Morphol. 265, 87–101 (2005) . 15880507 10.1002/jmor.10339

[b26] Vorobyeva E. I., Antipenkova T. P., Kolobayeva O. V. & Hinchliffe J. R. Some peculiarities of development in two populations of *Salamandrella keyserlingii* (Hynobiidae, Caudata). Russ. J. Herpetol. 7, 115–122 (2000) .

[b27] Wake D. B. & Shubin N. Limb development in the Pacific giant salamanders, *Dicamptodon* (Amphibia, Caudata, Dicamptodontidae). Can. J. Zool. 76, 2058–2066 (1998) .

[b28] Wong C. J. & Liversage R. A. Limb developmental stages of the newt *Notophthalmus viridescens*. Int. J. Dev. Biol. 49, 375–389 (2005) . 15968583 10.1387/ijdb.041910cw

[b29] Kumar A., Gates P. B. & Brockes J. P. Positional identity of adult stem cells in salamander limb regeneration. C. R. Biol. 330, 485–490 (2007) . 17631442 10.1016/j.crvi.2007.01.006

[b30] Gardiner D. M., Muneoka K. & Bryant S. V. The migration of dermal cells during blastema formation in axolotls. Dev. Biol. 118, 488–493 (1986) . 3792618 10.1016/0012-1606(86)90020-5

[b31] Kragl M. *et al.* Cells keep a memory of their tissue origin during axolotl limb regeneration. Nature 460, 60–65 (2009) . 19571878 10.1038/nature08152

[b32] Guimond J. C. *et al.* BMP-2 functions independently of SHH signaling and triggers cell condensation and apoptosis in regenerating axolotl limbs. BMC Dev. Biol. 10, 15 (2010) . 20152028 10.1186/1471-213X-10-15 PMC2829471

[b33] Bandyopadhyay A. *et al.* Genetic analysis of the roles of BMP2, BMP4, and BMP7 in limb patterning and skeletogenesis. PLoS Genet. 2, e216 (2006) . 17194222 10.1371/journal.pgen.0020216 PMC1713256

[b34] Benazet J. D. *et al.* *Smad4* is required to induce digit ray primordia and to initiate the aggregation and differentiation of chondrogenic progenitors in mouse limb buds. Development 139, 4250–4260 (2012) . 23034633 10.1242/dev.084822

[b35] Wake D. B. & Hanken J. Direct development in the lungless salamanders: what are the consequences for developmental biology, evolution and phylogenesis? Int. J. Dev. Biol. 40, 859–869 (1996) . 8877460

[b36] Brockes J. P. & Kumar A. Newts. Curr. Biol. 15, R42–R44 (2005) . 15668151 10.1016/j.cub.2004.12.049

[b37] Raspopovic J., Marcon L., Russo L. & Sharpe J. Modeling digits. Digit patterning is controlled by a Bmp-Sox9-Wnt Turing network modulated by morphogen gradients. Science 345, 566–570 (2014) . 25082703 10.1126/science.1252960

[b38] Hanken J. in The Origin and Evolution of Larval Forms eds Hall B. K., Wake M. H. 61–108Academic Press (1999) .

[b39] Harris R. N. in Tadpoles. The Biology of Anuran Larvae eds McDiarmid R. W., Altig R. 279–294Univ. Chicago Press (1999) .

[b40] Reiss J. O. The phylogeny of amphibian metamorphosis. Zoology 105, 85–96 (2002) . 16351859 10.1078/0944-2006-00059

[b41] Brockes J. P. in Salamanders in Regeneration Research. Methods and Protocols Vol. 1290, eds Kumar A., Simon A. 3–15Springer (2015) .

[b42] Wells K. D. The Ecology and Behavior of Amphibians Univ. Chicago Press (2007) .

[b43] Che R., Sun Y., Wang R. & Xu T. Transcriptomic analysis of endangered Chinese salamander: identification of immune, sex and reproduction-related genes and genetic markers. PLoS ONE 9, e87940 (2014) . 24498226 10.1371/journal.pone.0087940 PMC3909259

[b44] Carroll R. L. The palaeozoic ancestry of salamanders, frogs and caecilians. Zool. J. Linn. Soc. 150, 1–140 (2007) .

[b45] Frobisch N. B., Bickelmann C. & Witzmann F. Early evolution of limb regeneration in tetrapods: evidence from a 300-million-year-old amphibian. Proc. R. Soc. B 281, 201415502014. 10.1098/rspb.2014.1550 PMC4211449 25253458

[b46] Frobisch N. B., Carroll R. L. & Schoch R. R. Limb ossification in the Paleozoic branchiosaurid *Apateon* (Temnospondyli) and the early evolution of preaxial dominance in tetrapod limb development. Evol. Dev. 9, 69–75 (2007) . 17227367 10.1111/j.1525-142X.2006.00138.x

[b47] Schoch R. R. Amphibian Evolution. The Life of Early Land Vertebrates Wiley-Blackwell (2014) .

[b48] Cameron C., Beug S. & Tsilfidis C. Captive breeding of *Notophthalmus viridescens* through hormonal manipulation. Herpetol. Rev. 35, 257–259 (2004) .

[b49] Hayashi T. *et al.* Transcription activator-like effector nucleases efficiently disrupt the target gene in Iberian ribbed newts (*Pleurodeles waltl*), an experimental model animal for regeneration. Dev. Growth Differ. 56, 115–121 (2014) . 24329771 10.1111/dgd.12103

[b50] Dumond H. *et al.* Temporal and spatial *SOX9* expression patterns in the course of gonad development of the caudate amphibian *Pleurodeles waltl*. J. Exp. Zool. 316B, 199–211 (2011) . 10.1002/jez.b.21390 21462314

[b51] Aldea D. *et al.* Evolution of the vertebrate bone matrix: an expression analysis of the network forming collagen paralogues in amphibian osteoblasts. J. Exp. Zool. 320, 375–384 (2013) . 10.1002/jez.b.22511 23677533

[b52] Harland R. M. in Methods Cell Biol Vol. 36, eds Kay B. K., Peng H. B. 685–695Academic Press (1991) .1811161

[b53] Hama H. *et al.* Scale: a chemical approach for fluorescence imaging and reconstruction of transparent mouse brain. Nat. Neurosci. 14, 1481–1488 (2011) . 21878933 10.1038/nn.2928

