# Supplementary material for: An orphan gene is necessary for preaxial digit formation during salamander limb development
Source: Nat Commun. 2015 Oct 26;6:8684. doi: 10.1038/ncomms9684 (PMC4918474; doi:10.1038/ncomms9684)
Supplement: Supplementary Information — Supplementary Figures 1-8 [file ncomms9684-s1.pdf]

Supplementary Figure 1

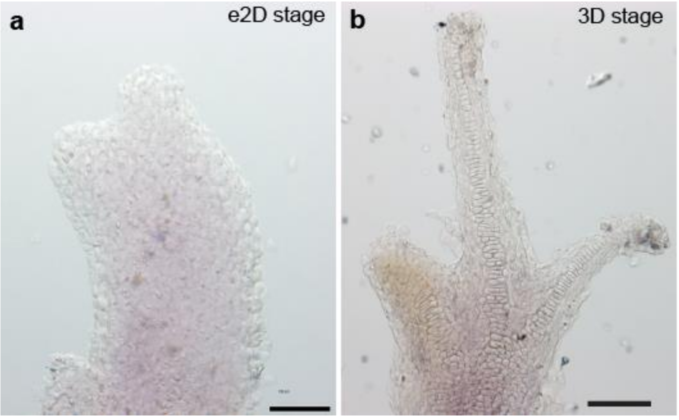

Preaxial dominance during salamander limb development. Panel **(a, b)** shows RNA *in situ* hybridisation on whole-mount limbs with *Sox9* sense probe as a control. Note the absence of reactivity with the sense probe in both cases. **(a)** e2D stage limb, (**b)** 3D stage limb. Scale bars, 100 µm.

Supplementary Figure 2

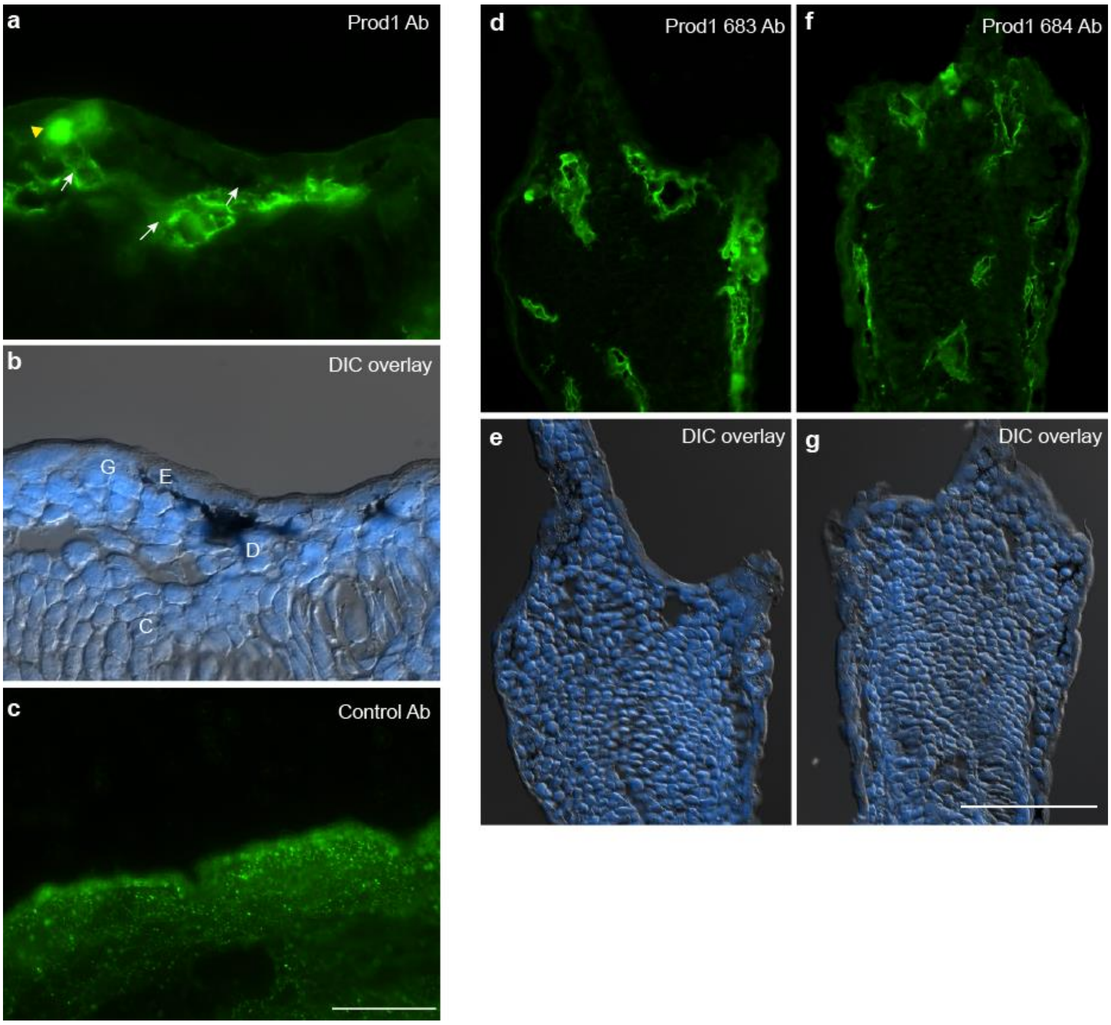

Prod1 expression in developing limbs. Panel (**a-c**) denotes mesenchymal expression of Prod1 protein during limb differentiation. (**a**) High magnification image showing Prod1 expression in dermal cells (arrowed) below the larval epidermis. A unicellular gland cell (yellow arrow) is also reactive to Prod1 protein. (**b**) Morphology of the limb section with a DIC image overlaying with nuclear staining. (**c**) Section of the limb reacted in parallel with concentration-matched non-specific polyclonal antibody as control. Scale bar, 50 µm.

Panel (**d-g**) represents longitudinal section of a 3-digit stage limb showing Prod1 protein reactivity. Contralateral limb sections from a larval newt were reacted with non-overlapping peptide antibodies 683 and 684 in parallel. (**d**) Prod1 683 antibody. (**e**) Morphology of the limb using differential interference contrast (DIC) and nuclear staining. (**f**) Prod1 684 antibody. (**g**) Corresponding morphology of the limb. C, cartilage; D, dermis; E, epidermis; G, unicellular gland. Scale bar, 100 µm.

Supplementary Figure 3

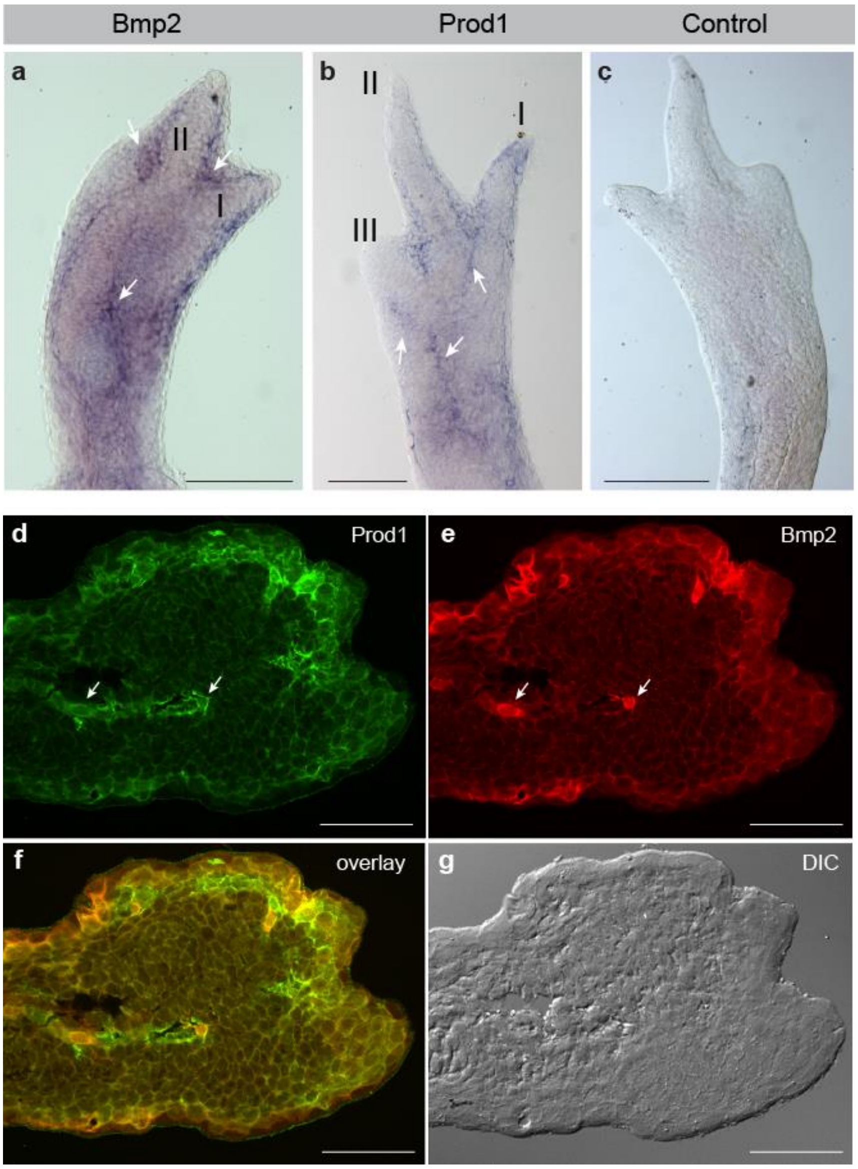

Early expression of Prod1 and Bmp2 in limb development. Panel (**a-c**) represents whole-mount RNA *in situ* hybridisation analysis at e3D stage limbs (n=5). (**a**) *Bmp2*, (**b**) *Prod1*, (**c**) Control sense probe to *Bmp2*. The arrows denote *Prod1* or *Bmp2* expressing cells. Scale bars, 100 µm.

Panel (**d**-**g**) represents longitudinal section of a limb at early 2-digit stage showing Prod1 protein reactivity in mid-line cells (**d**) and Bmp2 protein reactivity in (**e**). The cells arrowed in (d**)** and (e**)** co-express the proteins as shown in overlay (f). The image in (**g**) shows a DIC overlay to reveal the morphology of the limb section. (n=6). Scale bars, 50 µm.

Supplementary Figure 4

Case 1

```
TCAGCTACAAGACATGATGCTTCTACCACTCTCCTTGTTTCTGGTGGCATGCCTGCA
TCAGCTACAAGACATGATGCT---------CTCCTTGTTTCTGGTGGCATGCCTGCA
TCAGCTACAAGACATGAT--------------CCTTGTTTCTGGTGGCATGCCTGCA
TCAGCTACAAGACATGATGCTTCTACC-------TTGTTTCTGGTGGCATGCCTGCA
TCAGCTACAAGACATGATGCTTCT-----------TGTTTCTGGTGGCATGCCTGCA
TCAGCTACAAGACATGATGCTTCT------------GTTTCTGGTGGCATGCCTGCA
TCAGCTACAAGACATGAT-----------------TGTTTCTGGTGGCATGCCTGCA
TCAGCTACAAGACATGATGCTTCT--------CCTTGTTTCTGGTGGCATGCCTGCA
TCAGCTACAAGACATGATGCTTCTACC--TCTCCTTGTTTCTGGTGGCATGCCTGCA
TCAGCTACAAGACATGATGCTT--------------GTTTCTGGTGGCATGCCTGCA

TCAGGTACAAGACATGATGCTTGTACCACACTCTCCTTGTTTCTGGTGGCATGCCTGCA
```

Case 2

```
TCAGCTACAAGACATGATGCTTCTACCACTCTCCTTGTTTCTGGTGGCATGCCTGCA
TCAGCTACAAGACATGATGCTTCTACC--TCTCCTTGTTTCTGGTGGCATGCCTGCA
TCAGCTACAAGACATGATGCTTCTACCA---TCCTTGTTTCTGGTGGCATGCCTGCA
TCAGCTACAAGACATGATGCT-----------CCTTGTTTCTGGTGGCATGCCTGCA
TCAGCTACAAGACATGATGCTTCT-----------TGTTTGTGGCGGGATGCCTGCA
TCAGCTACAAGACATGATGCTTCTACC-CTCTCCTTGTTTCTGGTGGCATGCCTGCA
TCAGCTACAAGACATGATGCTTCT--------CCTTGTTTCTGGTGGCATGCCTGCA
TCAGCTACAAGACATGATG------------------TTTCTGGTGGCATGCCTGCA
TCAGCTACAAGACATGATGCTT--------------GTTTCTGGTGGCATGCCTGCA

TCAGCTACAAGACATGATGCTTCTACCAACTCTCCTTGTTTCTGGTGGCATGCCTGCA
TCAGCTACAAGACATGATGCTTCTACCAACTACTCTCCTTGTTTCTGGTGGCATGCCTGCA
```

Case 3

```
TCAGCTACAAGACATGATGCTTCTACCACTCTCCTTGTTTCTGGTGGCATGCCTGCA
TCAGCTACAAGACATGATGCTTCT------------------GGTGGCATGCCTGCA
TCAGCTACAAGACATGATGCTTC-------CTCCTTGTTTCTGGTGGCATGCCTGCA
TCAGCTACAAGACATGATGCTTCTA---------TTGTTTCTGGTGGCATGCCTGCA
TCAGCTACAAGACATGATGCTTCT--------CCTTGTTTCTGGTGGCATGCCTGCA
TCAGCTACAAGACATGATGCTTCTA-------CCTTGTTTCTGGTGGCATGCCTGCA
```

Mutational analysis of Prod1 TALEN larval newts. Three examples of sequences found at the exon 1 site after TALEN-mediated disruption. The mutant limbs were arrested in growth and showed absence of Prod1 protein in sections of the limb. Detailed analysis of the mutants (see Methods) shows extensive deletions in all the samples and insertions in two cases (1 and 2). The deletions are indicated by dotted lines, whereas, insertions are specified in red letters.

Supplementary Figure 5

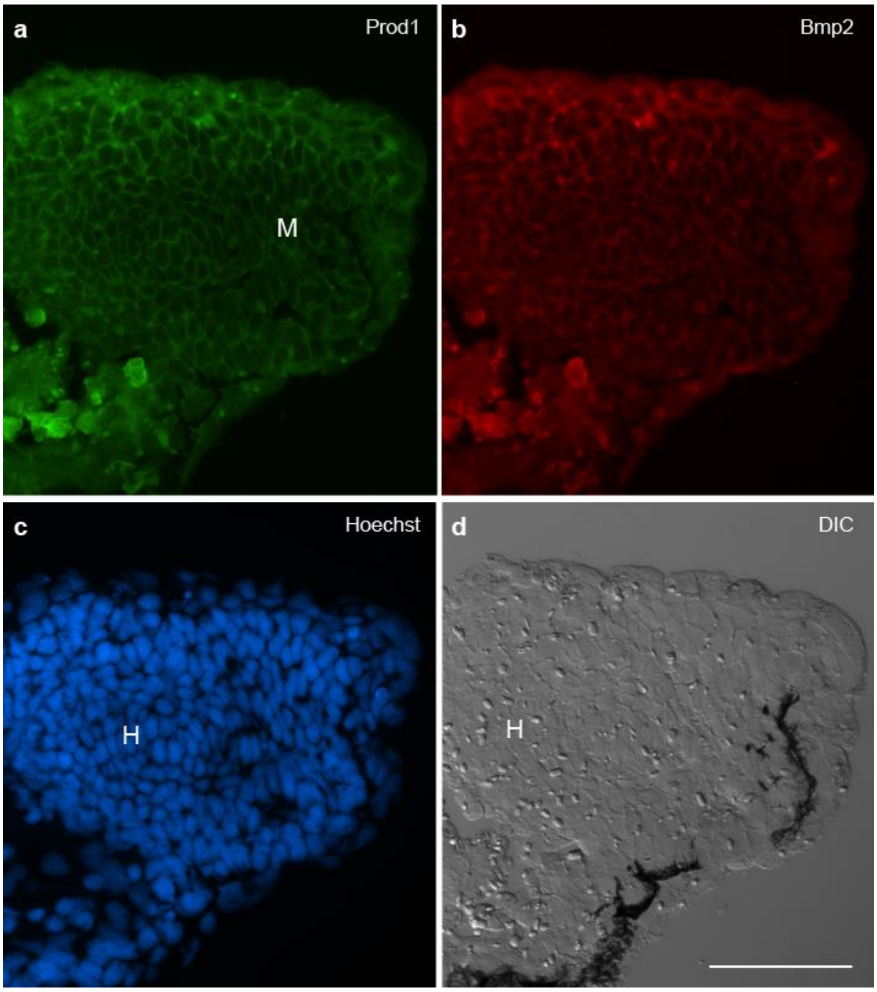

Absence of expression of Prod1 and Bmp2 in Prod1 TALEN limbs. Longitudinal section of a limb bud from a Prod1 TALEN disruptant showing the absence of the immunoreactivity of Prod1 protein (**a**) and Bmp2 (**b**). (**c**) Nuclear staining of limb bud cells. (**d**) Morphology of the limb bud using differential interference contrast (DIC) microscopy. Condensation of the cartilage denoting the formation of humerus is visible in of the proximal limb bud (n=8). H, humerus; M, mesenchyme. Scale bar, 100 µm.

Supplementary Figure 6

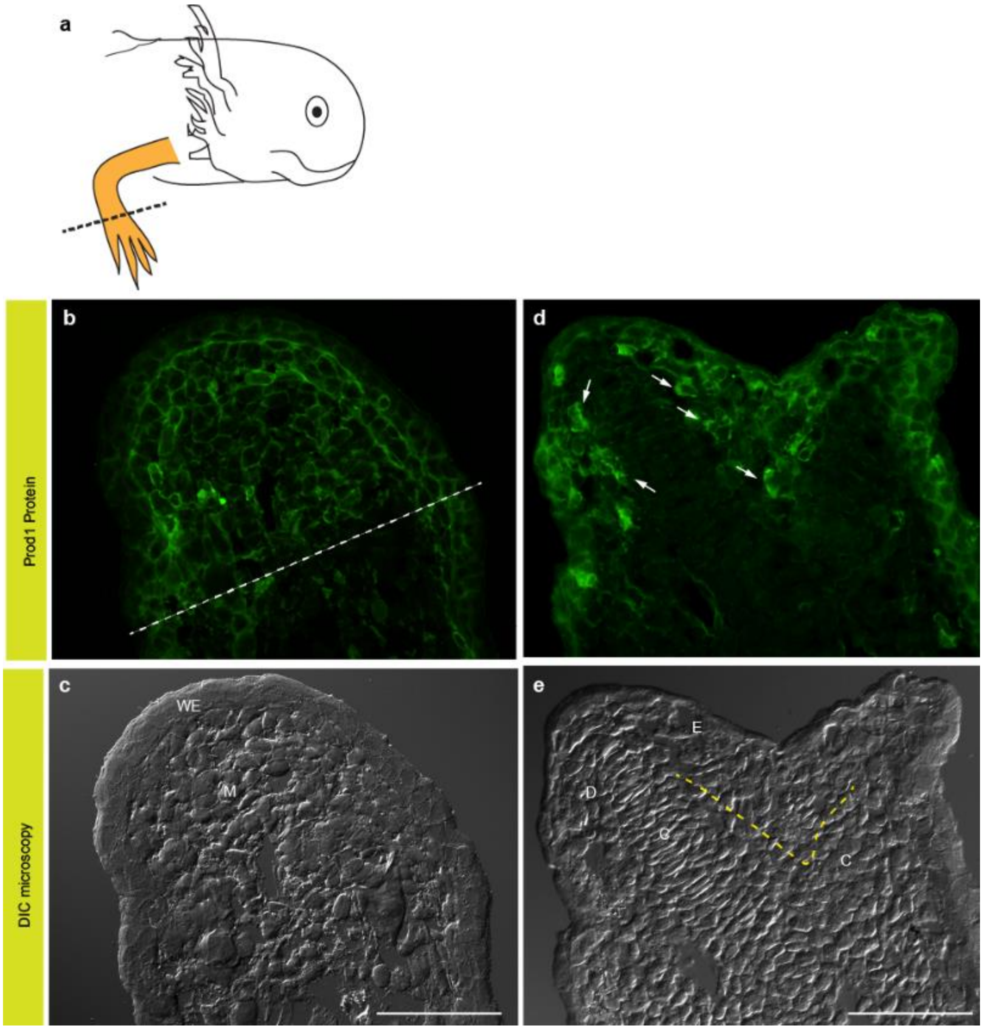

Limb regeneration in larval newts. (**a**) Schematic diagram outlining experimental design. The larval newt limbs were amputated during 4D stage of growth at mid-zeugopodium and were allowed to regenerate. (**b**) Limb blastema at 7d post-amputation showing reactivity of Prod1 protein. The dotted line indicates amputation plane. (**c**) Corresponding morphology of the limb section. (**d**) A longitudinal section of 2-digit limb showing Prod1 reactivity (arrowed) in mesenchymal cells beneath the larval epidermis. (**e**) Morphology of the regenerate. The yellow dotted line indicates the boundary between the cartilage cells and mesenchymal compartment. C, cartilage; D, dermis; E, epidermis; M, mesenchyme; WE, wound epithelium. (n=6 in both cases). Scale bars, 100 µm.

Supplementary Figure 7

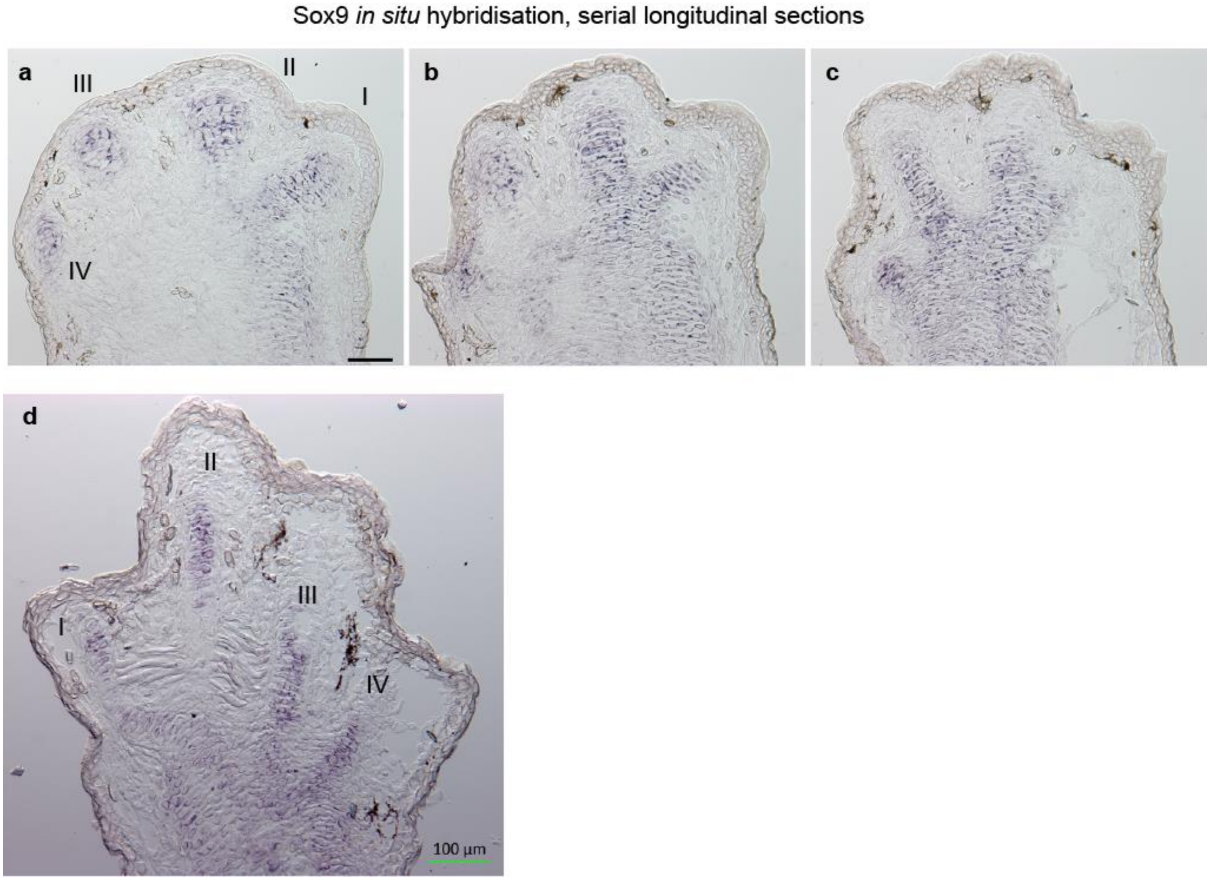

Limb regeneration in post-metamorphic (eft stage) newts. Panel **(a-c)** represents serial longitudinal sections from a regenerating limb (related to Fig. 5c) of an eft stage newt showing *Sox9* expression in the digits by in situ hybridisation. The regenerating limb is tapered along the dorso-ventral plane, therefore, the digits are not represented in a single plane of the section. (**d**) Another example of a regenerating limb section showing *Sox9* expression in digits I-IV. Scale bars, 100 µm.

Supplementary Figure 8

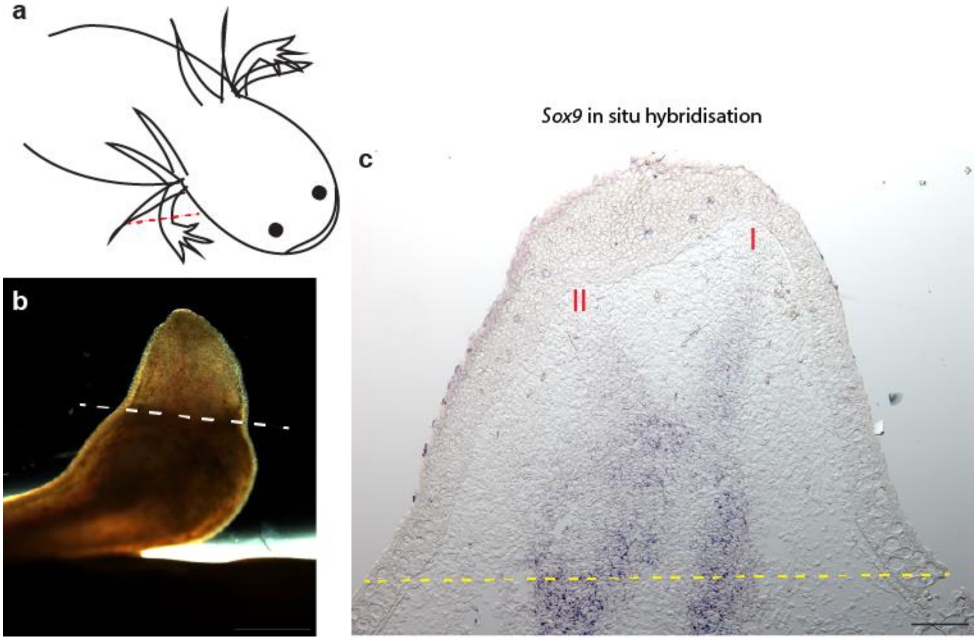

Limb regeneration in axolotls. (**a**) Schematics of the experimental design. The forelimbs of the paedomorphic axolotls were amputated at mid-zeugopodium and were allowed to regenerate. The dotted line indicates the level of limb transection. (**b**) A representative forelimb regenerate at late blastema stage of growth. (**c**) Longitudinal section of a limb showing *Sox9* expression in the digit primordia I and II by RNA in situ hybridisation. (n=14), Scale bar, 100 µm.
